# [Retracted] MEG-3-mediated Wnt/β-catenin signaling pathway controls the inhibition of tunicamycin-mediated viability in glioblastoma

**DOI:** 10.3892/ol.2025.15162

**Published:** 2025-06-30

**Authors:** Xiangyu Cui, Dezhou Sun, Bin Shen, Xin Wang

Oncol Lett 16: 2797–2804, 2018; DOI: 10.3892/ol.2018.9048

Following the publication of the above paper, a concerned reader drew to the Editor's attention that the immunofluorescence assay data shown in Fig. 6E and F had apparently already appeared in a figure in a paper published in the journal *Oncotarget* that was written by different authors at different research institutes. Upon performing an independent analysis of the data in the Editorial Office, it came to light that western blot data shown in Figs. 2E, 2F and 3E, and the migration and invasion assay data shown in Fig. 2A-D, were also strikingly similar to data that had already appeared in previously published papers in different journals written by different authors at different research institutes.

Given that the abovementioned data had already been published previously in a range of different journals, the Editor of *Oncology Letters* has decided that this article should be retracted from the Journal. After contacting the authors, they accepted the Editor's decision to retract this paper. The Editor apologizes to the readership for any inconvenience caused.

